# The path less traveled: the non-canonical NF-κB pathway in systemic lupus erythematosus

**DOI:** 10.3389/fimmu.2025.1588486

**Published:** 2025-07-02

**Authors:** Hilary Montano, Irving Coy Allen, Christopher M. Reilly

**Affiliations:** ^1^ Department of Biomedical Sciences and Pathobiology, Virginia-Maryland College of Veterinary Medicine, Virginia Polytechnic Institute and State University, Blacksburg, VA, United States; ^2^ Edward Via College of Osteopathic Medicine, Blacksburg, VA, United States

**Keywords:** lupus, NF-κB, non-canonical, BAFF, NIK

## Abstract

Systemic Lupus Erythematosus (SLE) is an autoimmune disease in which autoantibody production and cytokine dysregulation leads to systemic organ and tissue damage that can result in mortality. Although various environmental, hormonal, and genetic factors can contribute to disease pathogenesis, the cause of this disease is not known. Traditional treatment for this disease is centered around limiting inflammation using a variety of immunosuppresants including glucocorticosteroids as well as other therapeutics including anti-malarial drugs. More recently, selective immunosuppresives and biologics including Belimumab, a BAFF monoclonal antibody, and Anifrolumab, a monoclonal antibody that selectively binds to type 1 interferon receptor (INFAR1) blocking the biological activity of type 1 IFN, have been used with various success. It should be noted that BAFF is of particular relevance as signaling through BAFFR is a well characterized mechansim for non-canonical NF-κB signaling. While the canonical NF-κB pathway has been well studied and reported, the role of the non-canonical NF-κB pathway has been less investigated as to its role in autoimmunity. This pathway has been implicated in influencing pro-inflammatory immune responses while also regulating lymphocyte development. In this review, we aim to provide clarity on the relationship between the non-canonical NF-κB pathway and the role it plays in pathogenesis of SLE. The objective of this review is to summarize recent findings of the relationship of this pathway in autoimmunity and, more specifically, in lupus pathogenesis.

## 1 Systemic lupus erythematosus

Autoimmune diseases are primarily defined by an overactive immune system leading to loss of self-tolerance. Thus, the body’s immune system begins to attack itself (1, 2). Diseases like rheumatoid arthritis, multiple sclerosis, and systemic lupus erythematosus (SLE) all stem from this type of immune dysregulation (1, 3). At present, the cause of SLE is unknown, and so there is no cure for this disease (4). Disease onset can be triggered by genetic, epigenetic, immunoregulatory, hormonal, and environmental factors, although there is no single factor that can be used to predict disease (4, 5). Serious comorbidities attributed to lupus include cardiovascular disease and end-stage kidney disease (4–6). In particular, more than half of lupus patients develop kidney disease (lupus nephritis) which is the leading cause of mortality among lupus patients (4, 7). Mortality of patients diagnosed with SLE is over 2 times higher than that of the general population (8). SLE affects anywhere from 20 to 150 cases per 100,000 people with a higher prevalence in women of childbearing age at a 9:1 ratio and continues to increase as diagnostic methods become more refined (4). Additionally, people of African, Hispanic, and Asian ancestry have greater rates and more severe symptoms compared to those of Caucasian ancestry (1, 4, 9). Treatment of this disease is centered around reducing symptoms through the use of anti-inflammatory drugs, like glucocorticosteroids, and other therapeutics like anti-malarial drugs or biologics (3, 10, 11).

Immunologic dysregulation is central to SLE pathogenesis, with immune cells exhibiting abnormal cytokine activity that drives the chronic inflammation and tissue damage characteristic of lupus (4, 5). Several pro-inflammatory cytokines like interferon-alpha (IFN-α), tumor necrosis factor-alpha (TNF-a), interleukin-6 (IL-6), and interleukin-1 (IL-1) are associated with patients in active disease states (5, 12). Cytokines including interleukin-17 (IL-17), interleukin-21 (IL-21), and B-cell activating factor (BAFF) have also been implicated in autoimmune dysregulation of B- and T-cell responses (4, 13). In particular, upregulated levels of BAFF promote a loss of self-tolerance and are associated with higher levels of autoantibodies (14–16). The role of BAFF in lupus pathogenesis is further underscored by the approval of selective biologic therapies, such as Rituximab (anti-CD20) and Belimumab (anti-BAFF) which are two overexpressed cytokines, alongside traditional treatments like NSAIDs and DMARDs (3, 6). Autoantibody production mediated by prolonged B-cell activation and proliferation is a key hallmark in disease pathogenesis (4, 5, 17). Aberrant T-cell activity has similarly been observed to contribute to disease pathogenesis. Importantly, T-cells interact with both B-cells and myeloid cells further boosting the pro-inflammatory profile that is observed in this disease. Increased IL-17 and IL-21 levels are associated with T-cell hyperactivity (18, 19). Dysregulated levels of T-cell subsets are consistently observed in lupus patients where T follicular helper cell levels are increased while CD8^+^ T cells and T regulatory cells are reduced (4, 20, 21). Appropriate development and differentiation can be influenced by cell signaling pathways which are dysregulated in SLE (22, 23). This highlights the importance of identifying signaling pathway dysregulation and the downstream effects it has on this disease. Overall, it is evident that both the innate and adaptive immune system can play a critical role in disease.

## 2 The NF-κB pathway

**Figure 1:**
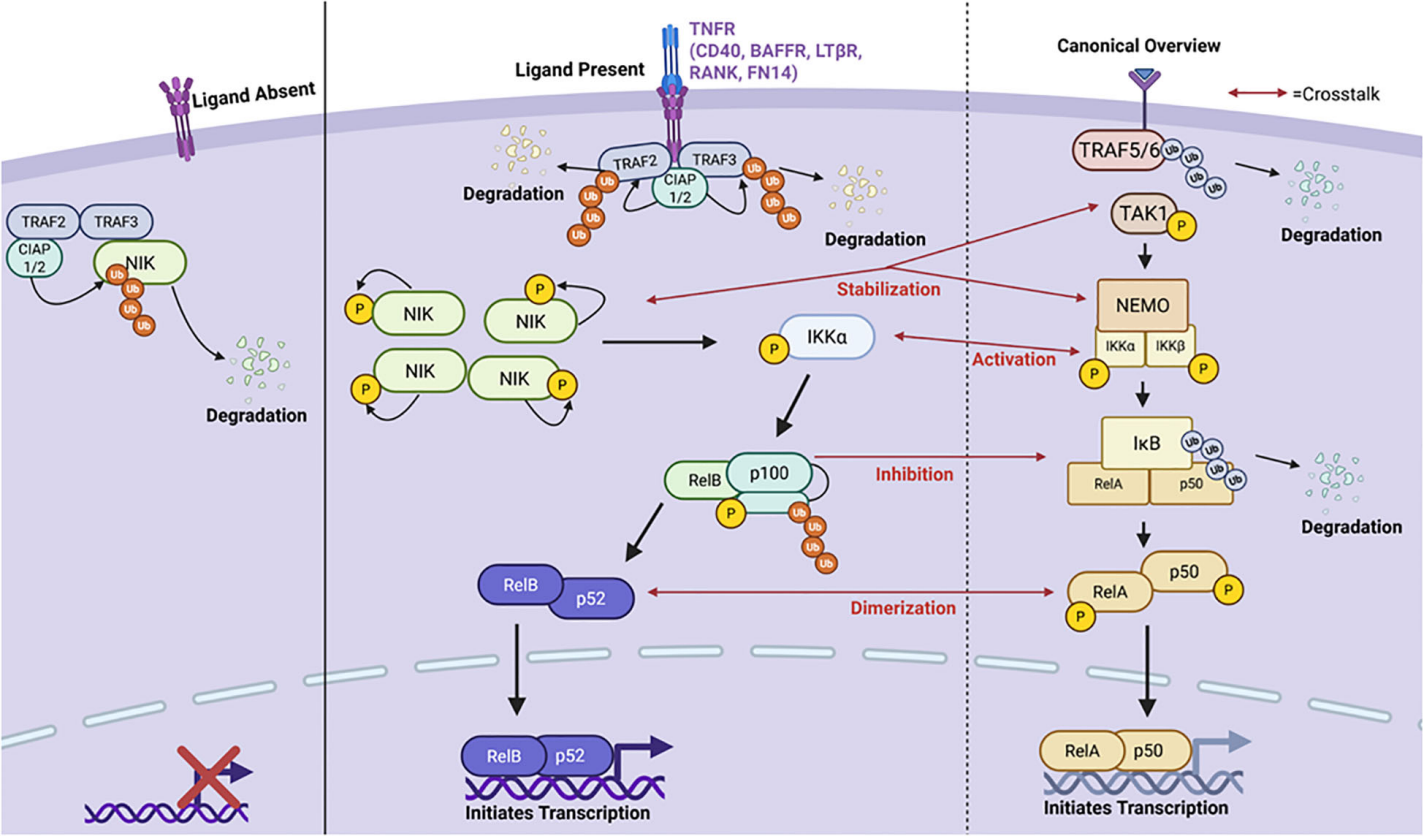
A brief summary of the canonical and non-canonical NF-κB pathways highlighting points of crosstalk between the two pathways. The non-canonical pathway is primarily activated by stimulation of the Tumor Necrosis Factor Receptor (TNFR). When the ligand is absent, a destruction complex consisting of TNFR Associated Factors 2 and 3 (TRAF2/3) and Cellular Inhibitor for Apoptosis Proteins 1 and 2 (cIAP1/2) binds and ubiquitinates NF-κB Inducing Kinase (NIK) degrading it in the cytoplasm and inhibiting pathway activation. However, upon ligand binding, the TNFR recruits the destruction complex enabling for TRAF2/3 ubiquitination and degradation by cIAP1/2. This allows NIK to accumulate and stabilize in the cytoplasm. NIK is autophosphorylated and can then phosphorylate Inhibitory-kB-Kinase α (IKKα). Upon its activation, IKKα cleaves p100 into p52 activating the RelB-p52 complex. RelB-p52 then translocates to the nucleus and initiates transcription of target genes. On the right panel is a brief overview of the canonical NF-κB signaling cascade with red arrows used to highlight points in which crosstalk between the two pathways occurs. Created in BioRender.com.

The canonical and non-canonical NF-κB pathways are complementary arms of NF-κB signaling that engage in distinct yet overlapping mechanisms to regulate immune homeostasis, inflammation, and lymphoid organogenesis. The canonical pathway is rapidly activated by pro-inflammatory signals such as TNF-α, IL-1, and TLR ligands, triggering the IKK complex composed of IKKα, IKKβ, and the regulatory subunit NEMO (IKKγ) (**Figure 1**) (24). This pathway can be specifically activated by pattern recognition receptors (PRRs), B cell receptors (BCRs), T cell receptors (TCRs), and by members of the tumor-necrosis factor receptors superfamily (TNFRSF) (25, 26). In this pathway, activation leads to phosphorylation of the inhibitor of nuclear factor-kappa B kinase (IKKβ) complex and the development of mature NF-κB subunits consisting of RelA/p65, c-Rel, and p50 which translocate to the nucleus and initiate transcription (26, 27). The canonical NF-κB pathway in autoimmunity and lupus has previously been reviewed by others (28–33). In contrast, the non-canonical pathway is selectively activated by a subset of TNF receptor family members (e.g., BAFF-R, CD40, LTβR) and depends on NF-κB-inducing kinase (NIK) and IKKα homodimers. NIK phosphorylates p100 (NF-κB2), which undergoes partial proteasomal processing to p52, allowing the formation and nuclear translocation of the p52/RelB heterodimer (**Figure 1**). Notably, NEMO, although not required for IKKα activation in the non-canonical pathway, can inhibit NIK accumulation by stabilizing a complex containing TRAF3, TRAF2, and cIAP1/2 that promotes NIK degradation under basal conditions (34). Thus, NEMO serves as a critical molecular node influencing both arms of NF-κB signaling.

When no ligand is bound to the receptor, NF-κB inducing kinase (NIK), which accumulates in the cytoplasm, is proteosomally degraded by a “destruction complex” consisting of cIAP1, cIAP2, TRAF2, and TRAF3. However, when a ligand is bound, this prevents NIK degradation allowing it to accumulate in the cytoplasm. As NIK accumulates in the cytoplasm, it stabilizes and is activated via auto-phosphorylation. It then phosphorylates IKKα which then initiates p100-processing thereby triggering degradation into its mature form of p52 (26). The p52-RelB heterodimer is then able to translocate to the nucleus and initiate transcription of target genes like B-cell Activating Factor (BAFF), CXCL13, and more (**Figure 1**) (35). This pathway is known to regulate a variety of processes including B cell maturation, secondary lymphoid development, cytokine and chemokine expression, and more (26).

Crosstalk between canonical and non-canonical pathways is particularly relevant in the context of SLE where dysregulation of feedback loops contributes to aberrant immune activation. In healthy cells, NIK stabilization is tightly controlled by TRAF3-mediated degradation, but excessive canonical signaling can influence this process through NEMO- and TRAF-mediated feedback, altering the NIK degradation machinery (36–38). Furthermore, RelB and NF-κB2 (p100/p52) are part of a transcriptional feedback loop where RelB can upregulate p100 expression, while p100 acts as an IκB-like inhibitor that sequesters both RelB and canonical dimers such as RelA in the cytoplasm (39, 40). This interdependency becomes pathogenic in SLE, where impaired processing of p100 or overexpression of RelB may lead to prolonged nuclear retention of NF-κB dimers and sustained transcription of inflammatory genes (41). Evidence suggests that aberrant BAFF signaling and dysregulated p100/RelB feedback contribute to hyperactive B cells, T cell dysfunction, and in lupus-prone mice and human SLE patients (42–45). These findings underscore the importance of balanced NF-κB signaling and suggest that targeting these inter-pathway checkpoints may provide therapeutic benefit in autoimmune disease. Currently, there are no direct pharmacologic agents known to selectively activate the non-canonical pathway. Limited publications explore pharmacological agents which can target this pathway. In a paper by Pache et al, they reported that small-molecular antagonists (SMAC) induced pathway activation through cellular inhibition of the apoptosis proteins, cIAP1 and cIAP2, leading to stabilization of NIK. This study highlighted the role of this pathway in HIV-1 latency reversal as a potential therapeutic in targeting latent viral reservoirs (46). There is evidence demonstrating independent regulatory roles of non-canonical NF-κB factors. For example, NIK has an alternative function in which it contributes to TNFR1-mediated RIP1-dependent apoptosis (47). Although this has been observed to be independent of non-canonical signaling, it was studied in mice depleted of proteins essential to the aforementioned “destruction complex”, another key factor in non-canonical signaling preventing NIK from accumulating in the cytoplasm (47). Crosstalk among the NIK-IKK complex has been observed to result in some canonical NF-κB activation by non-canonical factors, as seen in TRAF3^-/-^ mice, further contributing to an inflammatory profile (48). Additionally, crosstalk through canonical NF-κB regulation of the synthesis of p100 has also been reported. This pathway involves the interaction between different immune cells leading to the formation of a RelA/p52 heterodimer. Both of which are a combination of products from the canonical and non-canonical pathway (49, 50). Moreover, the non-canonical p100 itself can inhibit canonical products like RelA and c-Rel DNA binding activity causing the non-canonical pathway to regulate the canonical pathway (51, 52). On the other hand, the canonical pathway has been suggested to *prime* cells in preparation for non-canonical activation through influencing expression of non-canonical receptors like CD40 (50). The canonical pathway can also play a role in NIK stabilization through the T3-T2-cIAP E3 complex which regulates the ubiquitination and proteasomal degradation of NIK (25). IkB proteins are key signaling molecules in both pathways in different configurations (IKKa, IKKB). This is yet another area in which activation of one pathway may influence the activation of the other (35, 49). Both IKKα and IKKβ are IκB kinases involved in the respective pathways and overlap in their function. This presents challenges in dissecting the precise biological contributions of each. Tight regulation of both pathways is required for one’s immune system to function properly. While these pathways are not functionally independent as there is cross-regulation and co-activation common, understanding the contribution of each pathway for immune regulation and autoimmunity will allow for the development for more targeted therapeutics.

## 3 Cell specific roles

SLE disease is primarily caused by dysregulation of immune cells and the production of autoantibodies. This activity can be linked to aberrant activation of immunoregulatory protein signaling pathways, including the non-canonical NF-κB pathway. This pathway has been widely studied across multiple cell types within both the innate and adaptive immune systems; highlighting its distinct contributions to immunity and inflammation. Unlike the canonical NF-κB pathway, which responds to a broad range of inflammatory stimuli, the non-canonical pathway is activated by specific receptors, including BAFFR in B cells, CD40 in dendritic cells, and LTβR in stromal cells, leading to unique downstream effects on immune function. In the adaptive immune system, research on B and T cells has shown that the non-canonical pathway supports B-cell survival, maturation, and antibody production, while influencing T-cell differentiation and activation in response to antigen presentation (53, 54). Studies have also explored the role this pathway may play in dendritic cells. It has been revealed that non-canonical NF-κB signaling shapes cytokine production, antigen presentation, and inflammatory responses. This pathway’s role in stromal and endothelial cells also highlights its involvement in lymphoid tissue organization, crucial for the coordination of immune responses. Here, we will summarize the findings of the cell-specific ways in which the non-canonical pathway modulates immune balance and contributes to inflammation. Understanding cell specific mechanisms of non-canonical NF-κB signaling could provide a more comprehensive picture of how therapeutics may be developed to specifically treat autoimmune and inflammatory diseases.

### B cells

B cells are a key member of the adaptive immune system; however, dysregulation of these cells leads to human pathologies like autoimmunity and cancer (55). Lupus is often considered a B cell mediated disease due to their contribution to the chronic inflammatory state by dysfunctional overactivation and production of autoantibodies (4). The dysregulation of B cells is typically attributed to the failure of tolerance checkpoints, but other factors can further contribute to this. Studies have shown that autoreactive B cell activation is also be facilitated by diminished B cell receptor (BCR) signaling and aberrant cytokine signaling, such as IFNγ which can initiate B cell differentiation and activation (4, 56, 57). IFNγ is known to upregulate BAFF, a molecule also notably involved in B cell survival at different developmental stages (56). While checkpoints are present in B cell development, their failure along with confounding factors can prime the immune system in which overactive B cells and autoantibodies thrive.

**Figure 2:**
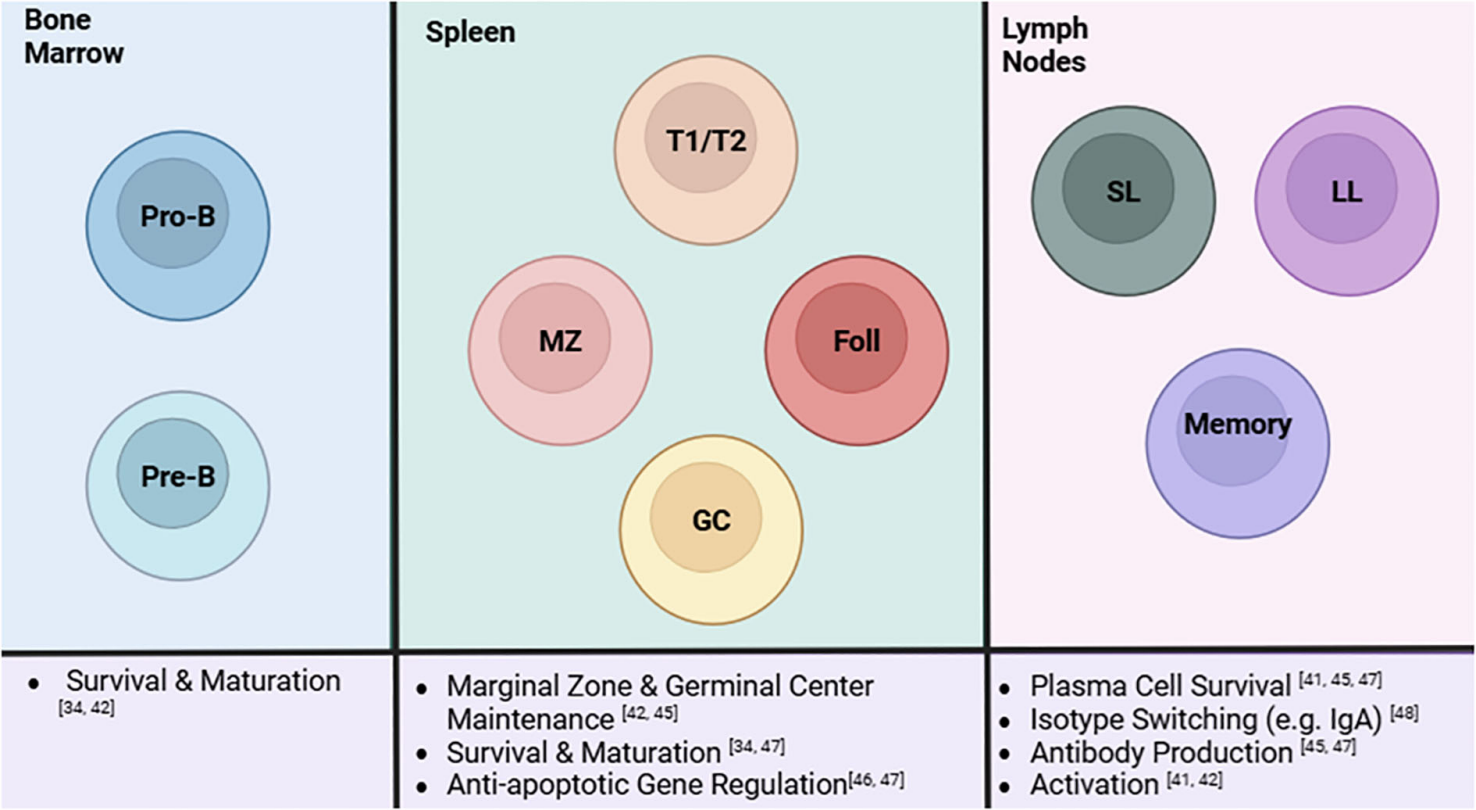
A summary of the role of the non-canonical NF-κB pathway in B cells and its influences at different stages of B cell development. The non-canonical pathway influences B cells at each stage in its development. Progenitor B (Pro-B) and precursor B (Pre-B) cells in the bone marrow rely on this pathway for survival and maturation. As they develop into the next stages at the spleen, the non-canonical pathway regulates survival, differentiation, and maturation of transitional 1/transitional 2, marginal zone, follicular, and germinal center B cells. MZ and GC B cells are reduced in the absence of the pathway. Additionally, these cells do not exhibit normal apoptotic activity when the pathway is depleted. This extends to B cell activity in the lymph nodes, specifically short-lived (SL) and long-lived (LL) plasma cells and memory B cells. The non-canonical pathway affects plasma cell survival, isotype switching, antibody production, and activation of these cell subsets. Created in BioRender.com.

B cells go through developmental stages starting in the bone marrow to the spleen and ending in the lymph nodes or periphery (58). B cell development is highly regulated, so much so that they are required to survive multiple checkpoints before they are able to migrate to the spleen (59). Various studies have looked into the role of the non-canonical pathway on B cells. While methods may vary between global and cell-specific deletion of a non-canonical pathway stimulator, regulator, or product, it has been consistently found that this pathway is active throughout immature and mature stages of B cell development (**Figure 2**) (60–66). NIK KO mice have been observed to have similar B cell deficiencies as BAFF deficient and CD40L/CD40 deficient mice including reduced proliferation and impaired germinal center development (60, 65). Along these lines, sanroque mice treated with an Fn14 antibody experienced reduced B cell and plasma cell numbers (61).

As B cells develop and migrate from the bone marrow to the spleen, the non-canonical pathway appears to regulate many aspects in the immature and transitional stages (**Figure 2**). The non-canonical NF-κB pathway has been determined to mediate immature B cell survival through BAFF signaling (65). It has been reported that NF-κB2 deletion as well as IKK-α deletion in mice show a diminished immature B cell population. Sun and coworkers reported that BAFF signaling through this pathway is also responsible for survival of transitional B cells through its regulation of anti-apoptotic factors like BCL-2 and BCL-X (25). A recent study identifying a novel regulator to the non-canonical pathway has also recapitulated much of these findings. TRIM55, a protein involved in p100 processing, was conditionally knocked out in B cells and this process was found to regulate marginal zone and follicular B cell development as well as mature B cell activation (66). Furthermore, cell specific deletion in a murine autoimmune model also supported findings that this pathway is involved in antibody production and class switching with reduced autoantibody and IgA production (66). A major responsibility of B cells is antibody production which is essential in adaptive immunity through developing immune memory to efficiently combat foreign antigens but can be considered a double-edged sword in the context of autoimmunity. This system is greatly impaired upon deletion and inhibition of members of the non-canonical pathway like NIK, Fn14, and BAFFR as these mouse models experience reduced IgA levels and appear to have impaired antibody responses and class switching (35, 60, 61). On the other hand, when TBK1, a negative regulator of the non-canonical NF-κB pathway, was knocked out, overproduction of systemic IgA and autoantibodies were observed in these mice (67). A similar phenotype of autoantibody production was also seen in mice overexpressing BAFFR demonstrating the effects of aberrant non-canonical signaling possibly in the context of autoimmunity (63). Biologics, like Belimumab (BAFF inhibitor) and Rituximab (CD20 inhibitor), have been developed to specifically reduce the B cell hyperactivity associated with lupus and other autoimmune diseases (4, 56). Since BAFF is involved in the non-canonical and canonical pathway, it is important for researchers to measure canonical factors in response to targeting non-canonical factors. Therefore, evidence suggests a potential link between the regulation of this pathway and the dysregulated B cell activity associated with SLE.

### T cells

T cells are the other major half of our adaptive immune system. In SLE, abnormal T cell populations and overactive CD4+ T cell activity can contribute to the chronic inflammatory state observed in lupus (21). T cells develop in the thymus before migrating to peripheral organs including the spleen and lymph nodes while activated T cells are recruited to infection sites. In the thymus, naïve T cells undergo positive and negative selection to ensure proper MHC class functions as well as the deletion of any autoreactive cells (68). When T cells are being assessed for self-reactivity in the thymus, medullary thymic epithelial cells (mTECs) play a critical role as they are responsible for expressing peripheral-tissue antigens in the thymus (69). This prevents autoreactive T cells from escaping the thymus. There are reports that autoreactive T cells are regulated by the non-canonical NF-κB pathway; specifically, through CD40 and RANK signaling (70). This is evidenced by Aly/aly mice, which have a mutated non-functional *Nik* gene. These mice develop loss of self-tolerance due to impaired mTEC development (35). While NIK is known to have other functions, this phenotype is replicated in RelB and NF-κB2 deficient mice as well mice lacking in other non-canonical pathway molecules including IKKα and LTBR, insinuating that this is specifically due to the impaired non-canonical pathway activation (63, 71).

Importantly, autoreactive T cells specifically contribute to SLE pathogenesis (72). Otherwise, this pathway appears to be dispensable in naïve T cell activation (26). Thus, this impairment often leads to the development of autoimmune like symptoms due to the presence of autoreactive T cells.

**Figure 3:**
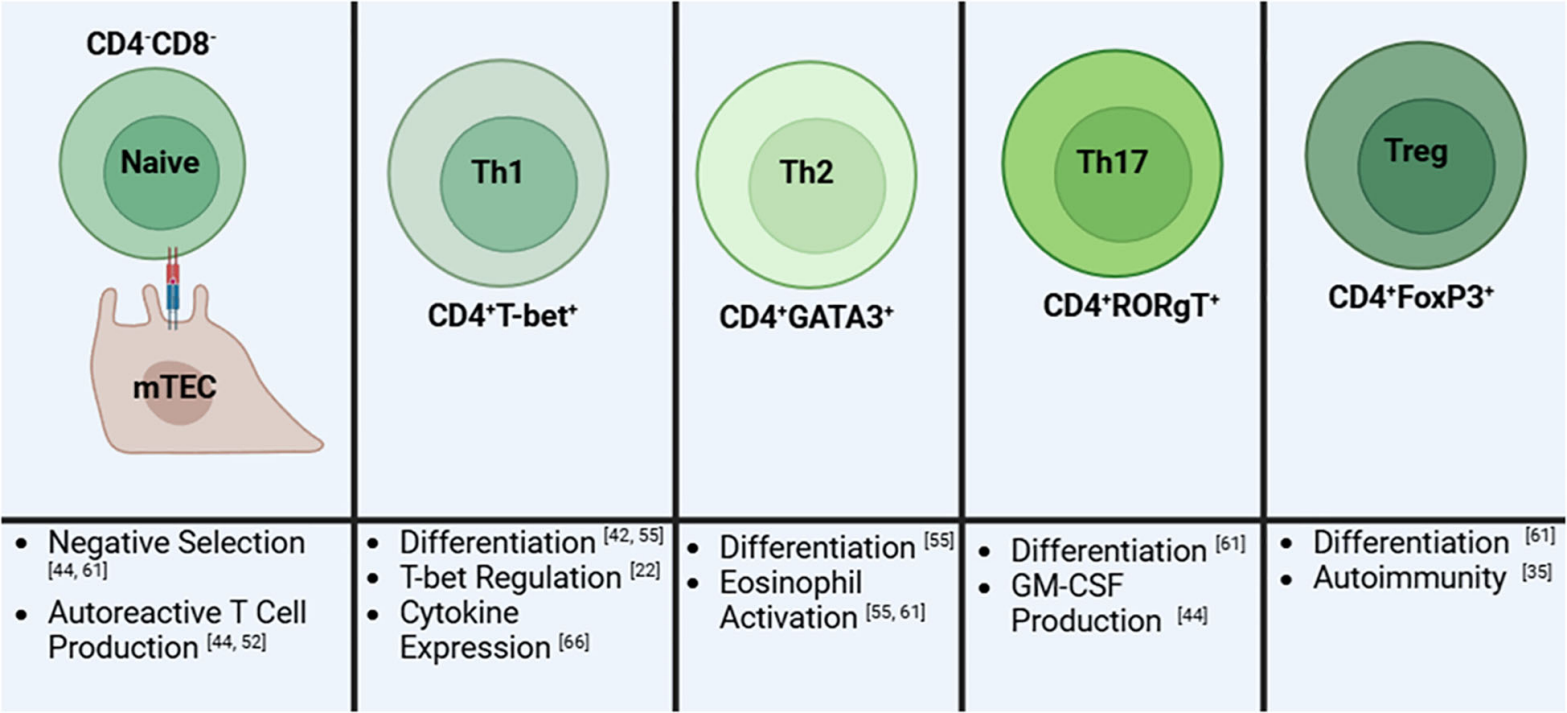
A summary of the role of the non-canonical NF-kB pathway on T cells and its influence on different T cell subsets. Early in development, naïve immature T cells will have impaired negative regulation which will result in an increase in autoreactive T cell production. Next, the differentiation into T helper 1 (Th1) cells is reduced due to decreased T-bet regulation. Th1 cells that develop then have altered cytokine expression. In contrast, T helper 2 (Th2) cells are elevated resulting in increased activation of eosinophils by these cells. Similarly, T helper 17 (Th17) cells are elevated leading to an increase in granulocyte-macrophage colony-stimulating factor (GM-CSF). T regulatory (Treg) cells are reduced in numbers thus directly contributing to autoimmunity. Created in BioRender.com.

T cells exit the thymus as either cytotoxic or helper T cells (68). While the non-canonical pathway doesn’t directly affect the cytotoxic T cell population, its dysregulation skews T cells towards a helper T cell population (**Figure 3**). Eden and coworkers showed that Lym1 mice, which produces a non-processible form of p100 preventing non-canonical activation, led to NF-κB2 deficiency and significantly greater T helper cells, double positive T cells, and double negative T cell populations in the periphery (73). Looking closer at these populations, later research revealed that RelB was involved in Th1 differentiation through its regulation of T-bet (63). Additionally, while NIK KO mice skew towards a Th2 phenotype characterized by IL-4 and IL-13 expression, Th1 cytokines including TNF, IFNγ, and IL-1β were also upregulated contributing to the hyper eosinophilic phenotype observed in these mice. This condition is likely due to the lack of lymphocytes in these mice due to global NIK deletion (74).

Still, it is evident that the loss of this pathway is directly affecting T cell differentiation, another point of contention in the context of lupus. Indeed, the independent depletion of NIK, IKKα, and NF-κB2 have further contributed to a T cell induced pro-inflammatory environment further driven by Th17 cell upregulation. The non-canonical product, p52, is associated with driving Th17 pathogenesis via GM-CSF production (75–77). In order to better understand the different roles of the canonical and non-canonical pathway in regulatory T cells (Tregs), Sato and colleagues (54) knocked out NF-κB1 and NF-κB2 in the MT-2 cell line and primary Tregs and found that only NF-κB2 deletion led to reduced FOXP3 expression and thus Treg activity. This reduction in Treg activity has also been studied in aly/aly mice illustrating further the importance of the non-canonical pathway on Treg survival (63). Tregs are of particular importance regarding SLE as these populations appear to be greatly reduced leading to uncontrolled activation of inflammatory T cells (19). The non-canonical pathway also affects the generation of T follicular helper cells through B cell activation. TWEAK/Fn14 and NIK inhibition are independently linked to decreased T follicular helper cells (62, 63, 77). This is likely due to depletion of ICOSL in B cells which is controlled by non-canonical signaling through BAFFR and CD40. In its absence, T follicular helper differentiation is reduced as it is dependent on the B cell activity driven by ICOSL (26, 62). Additionally, non-canonical dysregulation also leads to reduced effector memory T cells in the spleen which are responsible for providing faster responses against previously encountered antigens (62). While inhibition of NIK in T cells may lead to uneven subset distributions, overexpression leads to greater inflammatory and activation of the immune system largely due to the increased Th17 levels and overall T cell activation (27). Therefore, the proper regulation of this pathway is essential for normal T cell responsiveness and may play a role in promoting lupus-like symptoms.

### Dendritic cells

Dendritic cells are an antigen presenting cell (APC) and are essential for T-cell differentiation. Because of this, their function is important for activation of the adaptive immune response. They can be divided into conventional dendritic cells (cDC) and plasmacytoid dendritic cells (pDC). In SLE, cDC populations, which contribute to immune suppression, are reduced while pDCs, which contribute to pathogenic type I IFN production, are elevated (78). Several studies highlight the importance of the non-canonical pathway in dendritic cell activity. While aly/aly mice demonstrated that NIK is not required for dendritic cell development, it highlighted the importance of the non-canonical pathway in follicular dendritic cell formation in germinal centers (26). It was found that NIK and the non-canonical NF-κB pathway are essential for proper priming of CD8 cells and cross presentation. When NIK was deleted in CD11c+ cells, although lymphoid organs developed normally, they were unable to cross-prime naïve CD8+ T cells, even when treated with anti-CD40 to induce dendritic cell maturation. Furthermore, it was not due to improper MHC presentation which wasn’t affected in these mice (79).

NIK deficient dendritic cells are also associated with the inability to induce FOXP3 Treg expansion (77). Alternatively, when RelB was knocked out of dendritic cells in C57BL/6 mice, it resulted in spontaneous and elevated FOXP3 Treg development. Although NFκB2 deletion did not completely recapitulate this phenotypes, it can still be considered that part of this development is due its activation of the non-canonical pathway (80). As previously mentioned, crosstalk between the non-canonical protein p100 can inhibit canonical signaling activation. This activity has been specifically studied in dendritic cells in which p100 inhibits c-Rel leading to decrease expression of IL-23, a proinflammatory cytokine (51). Dendritic cells are also known to play a role in the gut microbiome. In fact, RelB deletion in dendritic cells led to greater levels of Tregs and IgA in the gut while also promoting eubiosis. Further studies demonstrated that elevated ncNF-κB activation in dendritic cells contributed to intestinal inflammation (81, 82). Deka et al. established a relationship between dendritic non-canonical signaling to the WNT/beta-catenin signaling pathway then influencing retinoic acid synthesis. Highlights of this study include how this signaling axis directly affected the gut microbiome, T regulatory cell activity, and IgA secretion (81). This is particularly significant regarding SLE as a common condition of this disease is a “leaky gut,” in which the intestinal mucosal layer is reduced and promotes systemic inflammation (83). Additionally, T regulatory depletion is another hallmark of lupus which is suggested to be regulated in the gut by non-canonical signaling in dendritic cells (81). Therefore, non-canonical NF-κB pathway regulation in dendritic cells is important as overexpression is associated with pathogenic activity relevant to SLE pathogenesis.

## 4 Non-canonical NF-κB in lupus

The non-canonical NF-κB pathway has emerged as a significant player in the pathogenesis of SLE contributing to dysregulated immune responses and chronic inflammation characteristic of these conditions. Dysregulation of this pathway has been implicated in other autoimmune diseases, including Common Variable Immunodeficiency (CVID), eosinophilic esophagitis (EoE), colitis, rheumatoid arthritis (RA), and systemic lupus erythematosus (SLE). In fact, Durmus and colleagues have published their findings demonstrating a positive association for serum NF-κB and juvenile SLE (jSLE) suggesting its importance as a potential biomarker (84). While this study focused on the canonical RelA subunit, this subunit is known to also dimerize with p52 through crosstalk between the canonical and non-canonical pathway (**Figure 1**) (49).

Aberrant activation of the noncanonical NF-κB pathway can occur through various mechanisms, such as chronic exposure to inflammatory cytokines, dysregulated signaling by TNF family members, or genetic mutations affecting pathway components essential to cell specific activity (**Table 1**). Once activated, this pathway promotes the production of pro-inflammatory cytokines, chemokines, and other mediators that drive immune cell infiltration, tissue damage, and perpetuation of the autoimmune response (26). NFκB2/p100/p52 mutations preventing pathway activation have been associated CVID characterized by impaired antibody response and defective T cell activity (63). As previously mentioned, NIK KO mice often have enhanced eosinophil expression which primarily localizes to the esophagus. When compared to human EoE patients, both species show non-canonical NF-κB signaling dysregulation with genes associated with the pathway having significantly enhanced expression (74).

**Table 1:** The relationship between cell specific non-canonical NF-κB regulation and SLE dysregulation.

Cell Type	ncNF-κB Regulatory Roles	Pathogenic SLE Roles	References
B Cells	• Early stages of survival and maturation • Anti-apoptotic gene regulation • Activation • Isotype switching • Antibody production	• Abnormal development • Evasion of tolerance checkpoints • Hyperactivation • Dysregulation cell survival • Autoantibody production	(3, 6, 22, 34, 36, 41, 43, 45, 47, 48)
T Cells	• Checkpoint evasion • Cytokine expression • Th17 activation • Treg activation (autoimmunity)	Autoreactive T cell activation ↓ CD8 T cells ↑ CD4 T cells IFN-γ production ↑ Th17 ↓ Tregs	(4, 6, 19, 35, 55, 61, 66)
Dendritic Cells	• Mucosal inflammation • Eubiosis • IgA stimulation • Follicular DC development • Cross priming • Maturation	• Leaky gut • Dysbiosis • Pro-inflammatory cytokine production (IFN-α) • Self antigen presentation	(10, 22, 28, 48, 56, 60, 62, 63)

This table compares regulatory roles of the non-canonical NF-kB pathway described in this review to known pathogenic cell activity in SLE demonstrating a possible relationship between the two.

↓ is decrease and ↑ is increase.

Beyond lymphocyte-intrinsic roles, the non-canonical NF-κB pathway also contributes to lupus pathogenesis through effects on mucosal immunity. Aberrant NIK activation in intestinal epithelial and dendritic cells has been linked to breakdown of the gut barrier and the development of a leaky gut phenotype which is a known amplifier of systemic inflammation in SLE (67, 68). This supports the concept that SLE is not solely a lymphocyte-driven disease, but one involving systemic disruption of immune homeostasis across tissues. Studies have also demonstrated that epithelial NIK and the non-canonical pathway in the gut provide protection from sepsis and colitis in the DSS-induced colitis model. This was also previously observed regarding dendritic RelB and NFκB2 in their influence of Tregs and IgA which altogether with the B-catenin/WNT signaling pathway influenced colitis symptoms phenotypically and at a transcriptional level demonstrating therapeutic potential in targeting both the non-canonical pathway and downstream at the B-catenin/WNT signaling pathway (81). In sum, elevated NIK activation is associated with more severe colitis symptoms, but depletion of NIK increases susceptibility. Regulated NIK in the gut supported commensal bacteria and protected against colitis (82). RA patients appear to have elevated levels of non-canonical stimulators in their synovial fluid, including CD40, BAFF, and RANKL, all of which can possibly contribute to inflammation observed in this disease (77). Therefore, non-canonical NF-κB dysregulation may be responsible for various human pathologies, and its activation needs to be tightly regulated.

Targeting of the non-canonical NF-κB pathway has emerged as a potential therapeutic strategy for SLE. As there are synthesized inhibitors of this signaling pathway, it may be possible to repurpose these therapeutics to potentially treat lupus including NIK SM1, XT2, and more (61, 85, 86). IKKβ Inhibitors modulate the canonical NF-κB pathway but also influence the non-canonical pathway. While both inhibitory-κB kinases (IKKs) IKKα and IKKβ play a central role in regulating the non-canonical and canonical NF-κB signaling pathway there is often overlapping effects making dissection of the functional roles of IKKα-mediated non-canonical NF-κB signaling versus IKKβ-driven canonical signaling pathway difficult to assess. Recently, Riley and coworkers reported on a novel series of IKKα inhibitors that selectively inhibit non-canonical NF-κB signaling. Similarly, the Mackay laboratory has also developed we specific IKKα inhibitor which also minimally affects the canonical pathway while successfully inhibiting non-canonical signaling (87). In their chronic lymphocytic leukemia studies, they observed that the use of this inhibitor reduces survival and proliferation of CD40L stimulated cells (88). This suggests potentially positive outcomes in the context of lupus since CD40L overexpression is a common characteristic in disease (89). The compounds (SU1261 and SU1349) have been used as pharmacological tools to interrogate the different signaling functions of IKKα and IKKβ in cells (90). In contrast, Bortezomib is a proteasome inhibitor approved for the treatment of multiple myeloma and mantle cell lymphoma which broadly inhibits NF-κB activation by preventing the degradation of IκB proteins, thereby affecting both the canonical and non-canonical pathways (91). While this inhibitor has shown promise in improving renal function in multiple myeloma patients, it may have limited impact for the treatment of lupus as there have been reports of toxicity with continued treatment (92). Another compound BAY 11–7082 has been reported to inhibit IkB kinase (IKK) activity and directly inhibit the NLRP3 inflammasome, leading to suppression of the NF-κB activation. Treatment of MRL/*lpr* mice showed decreased serum anti-dsDNA level and less renal immune complex deposition suggesting this may prevent lupus nephritis (93). Furthermore, in human blood dendritic cells, this compound inhibited nuclear translocation of IRF7 and IFN-α production (94). Furthermore, fusaproliferin is a mycotoxin that is naturally produced by the fungi genus *Fusarium* to protect itself against competing microorganisms (95). Fusaproliferin was shown to inhibit the activation of IKK thereby preventing degradation of IkB and decreasing phosphorylation of NF-κB. This reduced nuclear translocation of NF-κB leading to decreased IL-6, iNOS, and COX-2 in LPS-stimulated RAW 264.7 cells. However, this compound was not specific to IKK, suggesting other pathways were also inhibited (96). TANK-binding kinase 1 (TBK1) is a serine/threonine kinase belonging to the non-canonical inhibitor of nuclear factor-κB (IκB) kinase (IKK) family (97). TBK1 primarily mediates IRF3/7 activation and NF-κB signaling to regulate inflammatory cytokine production and the activation of innate immunity. The stimulator of interferon gene (STING) pathway contributes to the inflammatory response in lupus nephritis by upregulating *TBK1* and activating NF-κB signal pathway (98). As a non-canonical IκB kinases (IKK), TBK1 phosphorylates p100/NF-κB2, which is subsequently processed in the proteasome and released as a p52 subunit. TBK1 inhibitors are being explored for their roles in treating inflammatory diseases and cancer and may also serve as a therapeutic target for lupus (99). Overall, the non-canonical NF-κB pathway plays a crucial role in driving immune dysregulation and inflammation in SLE and more research is required to explore its capabilities as a possible therapeutic target in treatment this complex autoimmune disease.

## 5 Summary

The non-canonical NF-κB pathway is increasingly recognized as a critical regulator in the pathogenesis of systemic lupus erythematosus (SLE). Through selective activation by TNFR family receptors including BAFFR and CD40, B cell survival is enhanced, antibody class-switching increases, leading to autoreactive immune cells and lupus immunopathology. In addition to its role in lymphocyte function, non-canonical NF-κB signaling contributes to barrier dysfunction in the gut and promotes pro-inflammatory cytokine expression in dendritic cells, further exacerbating systemic inflammation. Emerging evidence from murine models and pharmacological studies highlight the therapeutic potential of selectively targeting this pathway. Inhibitors of NIK, IKKα, and TBK1 represent promising candidates for modulating this pathway and restoring immune homeostasis in lupus. Continued investigation into cell-specific regulation and signaling crosstalk is essential for refining targeted therapies and advancing clinical translation.

## Glossary

APC: antigen presenting cells
BAFF/R: B cell activating factor
BCL: B cell lymphoma
cIAP: cellular inhibitor of apoptosis protein
cDC: conventional dendritic cells
COX: cyclooxygenase
CVID: common variable immunodeficiency
DMARD: disease modifying antirheumatic drug
dsDNA: double stranded DNA
DSS: dextran sulfate sodium
EoE: eosinophilic esophagitis
Fn14: fibroblast growth factor-inducible protein 14
FOXP3: forkhead box p3
GC: germinal center
GM-CSF: granulocyte-macrophage colony-stimulating factor
IL: interleukin
ICOSL: inducible T cell costimulator
IFN (α: y), interferon
Ig: Immunoglobulin
INFAR: interferon alpha and beta receptor subunit 1
iNOS: inducible nitric oxide synthase
IκB: inhibitory kappa B
IKK: inhibitory-κB kinases
IRF: interferon regulatory factor
jSLE: juvenile systemic lupus erythematosus
LL: long lived
LPS: liposaccharide
LTBR: lymphotoxin beta receptor
MHC: major histocompatibility complex
MT-2: metallothionein-2
mTEC: medullary thymic epithelial cells
MZ: marginal zone
NEMO: nuclear factor-kappa B essential modulator
NIK: NF-κB inducing kinase
NF-κB: nuclear factor-kappa B
NLRP: nucleotide-binding oligomerization domain
NSAID: nonsteroidal anti-inflammatory drug
pDC: plasmacytoid dendritic cell
RA: rheumatoid arthritis
RANK/L: receptor activator of nuclear factor kappa B/ligand
SLE: systemic lupus erythematosus
SMAC: small-molecular antagonists
SL: short lived
TANK-TRAF: family member-associated NF-κB activator
Th (Th1/Th2/Th17): T helper
T-bet: T-box transcription factor
TBK1: tank-binding kinase 1
TLR: toll-like receptor
TNF (α): tumor necrosis factor
TNFR: tumor necrosis factor receptor
TRAF: tumor necrosis factor receptor associated factor
Treg: T regulatory
TRIM55: tripartite motif containing 55
TWEAK: tumor necrosis factor-like weak inducer of apoptosis
WNT: wingless/int-1
